# Elevated Serum Retinol Binding Protein 4 is Associated with the Risk of Diabetic Cardiomyopathy

**DOI:** 10.31083/j.rcm2304115

**Published:** 2022-03-24

**Authors:** Haihua Shan, Yanjing Ji, Haibo Gu, Hui Li, Jing Zhu, Yu Feng, Hao Peng, Tao You, Xiaosong Gu

**Affiliations:** ^1^Department of Anesthesiology, the Second Affiliated Hospital of Soochow University, 215004 Suzhou, Jiangsu, China; ^2^Department of Cardiology, the Second Affiliated Hospital of Soochow University, 215004 Suzhou, Jiangsu, China; ^3^Department of Endocrinology, the Second Affiliated Hospital of Soochow University, 215004 Suzhou, Jiangsu, China; ^4^Department of Epidemiology, School of Public Health, Medical College of Soochow University, 215123 Suzhou, Jiangsu, China; ^5^Department of Hematology, the First Affiliated Hospital of Soochow University, 215006 Suzhou, Jiangsu, China

**Keywords:** diabetic cardiomyopathy, retinol binding protein 4, diabetes mellitus, risk factor

## Abstract

**Background:**

Retinol binding protein 4 (RBP4), a biomarker for insulin resistance in type 2 diabetes (DM), is increased in heart failure. This case-control study aims to determine the association between serum RBP4 levels and diabetic cardiomyopathy (DCM).

**Methods:**

Demographic and clinical data were obtained from 245 DM patients and 102 non-diabetic controls. RBP4 levels were measured using ELISA. The association between RBP4 and DCM was evaluated using multivariate logistic regression and restricted cubic splines (RCS) in DM patients.

**Results:**

We showed that serum RBP4 levels were higher in DCM patients than in DM patients without DCM or the controls. Multivariate analysis adjusted by age, gender, body mass index, diabetes duration, left ventricular ejection fraction, insulin treatment, triglycerides, low-density lipoprotein cholesterol, estimated glomerular filtration rate, diabetic retinopathy, diabetic nephropathy, diabetic neuropathy and log N-terminal proBNP showed a significant association between RBP4 and DCM (highest vs. lowest tertile OR 16.87, 95% CI: 6.58, 43.23, *p *< 0.001). RCS displayed a positive linear correlation between RBP4 levels and the risk of DCM in diabetes (*p* = 0.004). Adding RBP4 to a basic risk model for DCM improved the reclassification (Net reclassification index: 87.86%, 95% CI: 64.4%, 111.32%, *p *< 0.001).

**Conclusions:**

The positive association between serum RBP4 and DCM suggested the role of RBP4 as a potential diagnostic biomarker for distinguishing DCM in patients with DM.

## 1. Introduction

Type 2 diabetes mellitus (DM) was associated with an increased risk of any left ventricular systolic and diastolic dysfunction [1]. Diabetic cardiomyopathy (DCM) was initially described as a pathophysiological condition in which heart failure occurred in diabetic patients without coronary artery disease, hypertension, and valvular heart disease [2]. Epidemiological studies in the U.S. showed a prevalence of DCM is 9.3% in the general population, and 19–26% of diabetic patients suffered from heart failure [3]. Meanwhile, 16.9% of the diabetic patients had diabetic cardiomyopathy and 54.4% had diastolic dysfunction [1]. Mortality from heart failure is among the leading causes of death in patients with DM, constituting a worldwide health and economic burden [4, 5]. However, most of the patients with DCM may not have any overt symptoms or signs of cardiac dysfunction before progressing to symptomatic heart failure. There is an urgent need for reliable and available biomarkers for DCM detection, identify a suitable biomarker will help in the recognition and management of DCM [6]. Therefore, screening of DCM patients may facilitate the early intervention and individualized management and improve the cardiovascular prognosis of diabetic patients [7].

Retinol binding protein 4 (RBP4) is a secreted protein of 21-kDa that transports retinol (vitamin A) in the circulation [8]. The majority of RBP4 is produced in the liver and adipocytes where dietary retinoids are stored and cleared. RBP4 is secreted into the plasma as an RBP4–retinol complex that delivers retinol to extrahepatic tissues [9]. Recent evidence suggests that it may function as an adipokine associated with metabolic homeostasis and elevated RBP4 levels are associated with insulin resistance [10]. Transgenic overexpression of RBP4 or chronic RBP4 administration induces whole-body insulin resistance and RBP4 deletion improves insulin action in mice [11, 12]. Serum RBP4 level is also correlated with visceral adiposity, body mass index (BMI), dyslipidemia, inflammation, and incipient nephropathy in patients with DM [10, 13, 14]. Interestingly, clinical observations showed that increased circulating RBP4 was associated with chronic heart failure (CHF), and elevated serum RBP4 was correlated with a worse outcome in elderly patients with CHF [15, 16]. RBP4 was also associated with the severity of insulin resistance in patients with obesity, impaired glucose tolerance, or DM [17]. These findings suggested that RBP4 plays a pernicious role in the cardiovascular complication of diabetes.

The association of RBP4 with cardiac dysfunction and metabolic disorders suggested its potential as a biomarker in the diabetic population. However, the relationship between RBP4 and DCM remains unclear. Therefore, we performed this case-control study to evaluate the association of serum RBP4 concentrations with the risk of DCM in patients with DM.

## 2. Materials and Methods

### 2.1 Study Population

A total of 245 patients with DM admitted to the second affiliated hospital of Soochow University (Suzhou, Jiangsu, China) for diagnostic coronary angiography due to chest discomforts from January 2017 to December 2019 were consecutively enrolled in this study. 102 controls without DM were selected from a healthy population undergoing routine physical examination during the same period. DM was defined according to the criteria of the American Diabetes Association (hemoglobin A1c (HbA1c) level ≥6.5% and/or a fasting plasma glucose (FPG) ≥7.0 mmol/L) [18].

**Fig. 1.**
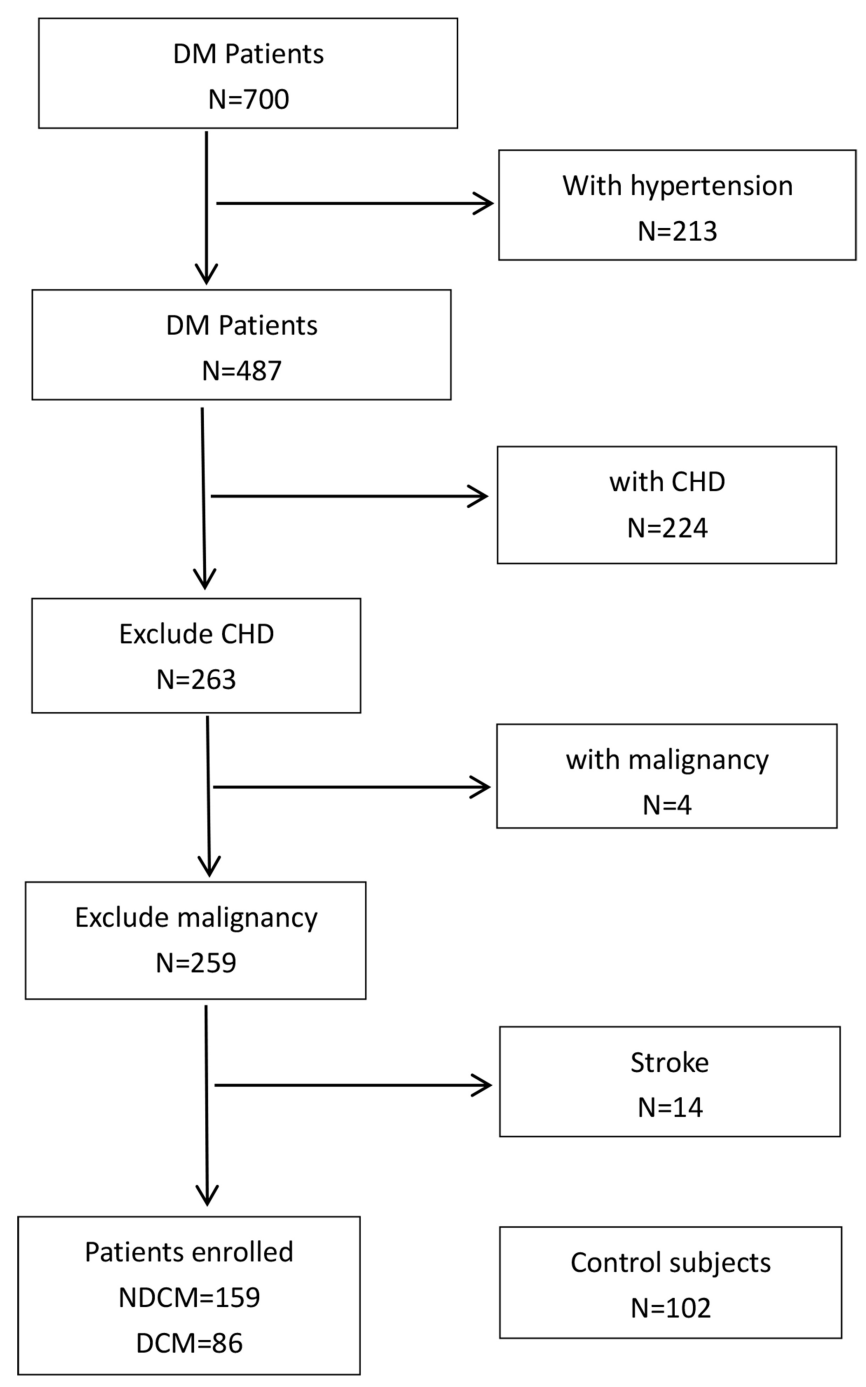
**Diagram showing patient flow throughout the trial**. Flow chart of study enrollment to illustrate the inclusion and exclusion criteria.

Coronary heart disease was defined as stenosis of at least 50% of the luminal diameter in at least one major coronary artery branch evaluated by coronary angiography [19]. Patients with coronary heart disease, idiopathic dilated cardiomyopathy, hypertension (SBP≥140 mmHg, DBP ≥90 mmHG), peripheral vascular disease, primary valvular heart disease, type 1 diabetics, chronic obstructive pulmonary disease, immunosuppressive therapy, renal failure (creatinine >2 mg/dL), malignant tumors and/or musculoskeletal conditions limiting exercise capacity such as rheumatoid arthritis were excluded (Fig. 1).

### 2.2 Data Collection

Demographic and clinical data including age, gender, BMI, systolic and diastolic blood pressure, diabetes duration, smoking habits, alcohol consumption, complications related to diabetes, insulin therapy and hypoglycemic drug treatment were recorded. Neuropathy was diagnosed after checking pin prick, vibration sense, ankle reflex, and knee reflex. Retinopathy was detected after examining microdots, blot hemorrhage, hard exudates, soft exudates, and new vessel formation. Nephropathy was noted upon finding urinary albumin in detailed urine reports.

Echocardiography was performed by a certified cardiologist on all participants. LV ejection fraction (LVEF) was obtained from 2D-images by manual tracing using the biplane Simpson method in 4- and 2-chamber views. Left ventricular diastolic dysfunction was determined by pulsed-wave Doppler examination of mitral inflow (before and during Valsalva maneuver) and by Doppler tissue imaging of the mitral annulus. We collected data on left atrial volume index, the early (E) and late (A) trans mitral inflow velocities, early diastolic velocity of the medial (septal) mitral annulus (e’), non-invasive assessment of left ventricular filling pressures (E/e’). Normal diastolic function was defined as an E/A between 0.75 and 1.5, normal left atrial volume index (<28 mL/$m^{2}$), and normal left ventricular filling pressure (E/e’ <10), Mild diastolic dysfunction included patients with an E/A of less than 0.75 and E/e’ <10. Moderate/severe diastolic dysfunction included patients with an E/A >1.5, left atrial volume index ≥28 mL/$m^{2}$, and E/e’ ≥10. Patients with a pseudo normal pattern were included in the moderate/severe diastolic dysfunction group as all had left atrial volume indices ≥28 mL/$m^{2}$ [20].

### 2.3 Definition of Diabetic Cardiomyopathy

DCM was diagnosed in patients according to the following criteria: (1) diabetes mellitus (2) moderate to severe diastolic dysfunction or LVEF <50%. Diastolic dysfunction was categorized according to the echocardiography-assessed progression of the diastolic disease (3) no history of coronary heart disease according to angiograph examination (4) No history of hypertension (SBP ≥140 mmHg, DBP ≥90 mmHg), (5) no history of significant valvular disease and (6) no history of congenital heart disease [1, 21].

### 2.4 Serum Sample Collection and Measurement

A total of 5 mL venous blood samples were collected from the study participants in the morning after a 12-h fasting period. After immediate centrifugation at 4 ^∘^C, aliquots were stored at –80 ^∘^C until analysis. Serum was diluted 1000-fold for RBP4 measurement because of the high concentration of RBP4 in human serum. RBP4 was measured using a Retinol Binding Protein-4 (Human) EIA kit (Phoenix Pharmaceuticals, Inc., Burlingame, CA, USA), with each value reported as the mean of duplicate measurements made on the same serum sample. The assay in this kit was linear for purified recombinant RBP4 from 3.12–31.4 ng/mL, test range was 0.1–1000 ng/mL, and intraassay and interassay coefficient of variability (CVs) were less than 5% and 14%, respectively.

N-terminal pro-B-type natriuretic peptide (NT-proBNP) was measured by biotin coupled anti-NT-proBNP antibody/streptavidin solid-phase chromatographic immunoassay with the ${\mathrm{StatusFirst}}^{\text{TM}}$ CHF NT-proBNP test device. Fasting plasma glucose (FBG), lipids, creatinine (Cr), blood urea nitrogen (BUN) were measured in the clinical laboratory. The estimated glomerular filtration rate (eGFR) was calculated using the CKD-EPI equation [22]. FBG was measured by an automated glucose oxidase method (Automatic Analyzer 2700, Olympus, Tokyo, Japan). HbA1c was measured by using the high performance liquid chromatographicanalysis (HPLC), Serum total cholesterol (TC), triglyceride (TG), high-density lipoprotein cholesterol (HDL), and low-density lipoprotein cholesterol (LDL-C) were measured by enzymatic methods using an autoanalyzer.

### 2.5 Statistical Analysis

Continuous variables with normal or skewed distributions are expressed as mean ± standard deviation (SD) or median (interquartile range [IQR]) and compared using the two-tailed student’s *t*-test or Mann-Whitney U test between two groups. Comparison of numeric variables between more than 2 groups was performed using the Kruskal Walli’s test with Dunnett’s post hoc analysis. The sample size was calculated using the Wilcoxon-Mann-Whitney Sample Size Calculation package in R with 2-sided alpha at 0.05, and power at 0.8. The normality of continuous variables was evaluated using the Shapiro-Wilk test. Serum RBP4 and NT-proBNP levels were normalized by log10 transformation (log RBP4 and log NT-proBNP). Categorical variables were presented as frequencies (percentages) and compared using Pearson’s chi-squared test. The correlations between serum RBP4 level and other variables were evaluated using Spearman’s correlation. The association between serum RBP4 and DCM in diabetic patients was assessed using logistic regression. The linear correlation between accentuating RBP4 and the risk of DCM was analyzed using the restricted cubic spline (RCS), with 3 knots placed at the 10th, 50th, and 90th percentiles of RBP4. Odds ratios and 95% confidence intervals (95% CIs) for upper quartiles of RBP4 regarding the reference lowest quartile was calculated using multivariable logistic regression adjusted for age, gender, diabetes duration, BMI, insulin treatment, LVEF, TG, LDL-C, eGFR, and log NT-proBNP. The improvement of discriminative ability and reclassification by log RBP4 beyond other DCM risk factors was evaluated using receiver operator characteristic and precision-recall curves. For model comparisons, continuous and categorical net reclassification index (NRI), and integrated discrimination index (IDI) were calculated. Two-tailed *p* values < 0.05 were considered statistically significant. NRI represented the incremental ability to accurately reclassify patients with DCM into higher risk categories and individuals without DCM into lower ones after RBP4 level was incorporated into the prediction models. IDI reflected the increase in difference of mean probability to predict DCM risk in cases with DCM than in controls, indicating whether the prediction model with additive RBP4 level had a better ability to distinguish cases from controls. Statistical analyses were performed using the PRROC, risk Regression, rms, caret, and final fit packages in R software (version 3.6.3, Vienna, Austria).

## 3. Results

### 3.1 Baseline Characteristics

**Fig. 2.**
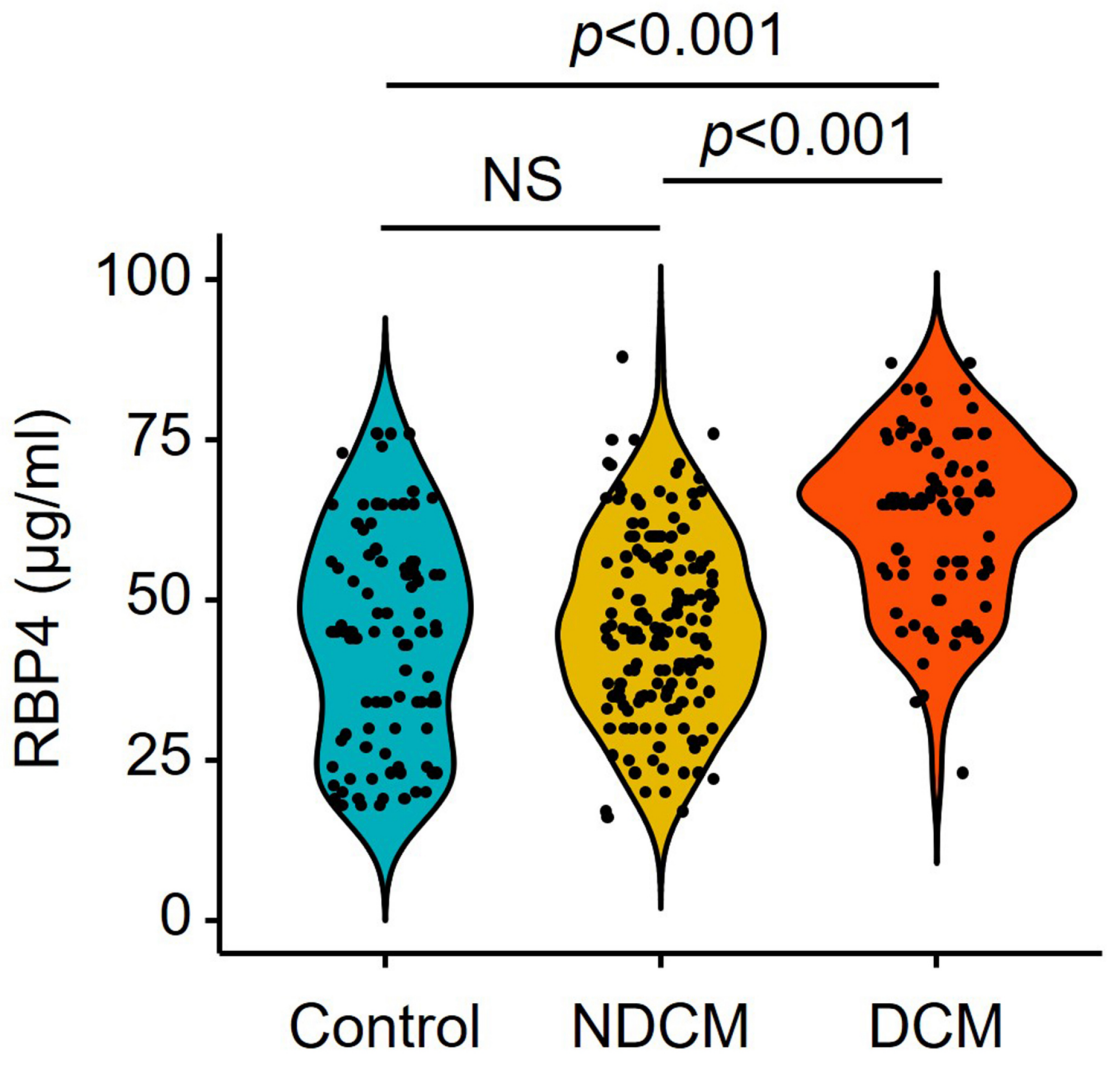
**Violin plot of serum RBP4 levels measured with enzyme-linked immunosorbent assay**. Control, participants without diabetes (n = 102). NDCM, diabetic patients without cardiac dysfunction (n = 159). DCM, patients with diabetic cardiomyopathy (n = 86).

The baseline characteristics of the controls, DM patients without cardiac dysfunction (NDCM), and DCM patients are shown in Table 1. Compared with the NDCM and control participants, the DCM patients were more likely to be older and had higher levels of TC, and LDL-C. The DCM group had lower eGFR and LVEF than the NDCM and control groups. Compared to the NDCM group, DCM patients showed longer diabetes duration, higher NT-proBNP levels, and a smaller proportion receiving insulin treatment. Serum RBP4 of DCM is higher in DCM than in NDCM (Fig. 2). The incidence rate of retinopathy and neuropathy are higher in DCM than NDCM (Table 1).

**Table 1.** **Baseline clinical, anthropometric and biochemical data**.

	Control (n = 102)	DM		*p*
		NDCM (n = 159)	DCM (n = 86)	
Male, n (%)	60 (60.0)	88 (55.3)	41 (47.7)	0.311
Age (IQR,years)	65.00 [60.00, 72.25]	66.00 [57.50, 74.00]	69.00 [65.00, 74.00]	0.027
BMI (IQR, kg/$m^{2}$ )	25.82 [23.41, 27.57]	25.44 (22.17, 27.72)	26.17(23.81, 28.00)	0.365
SBP (IQR, mmHg)	133.00 [123.75, 137.25]	132.00 [120.00, 138.00]	133.50 [125.00, 137.00]	0.949
DBP (IQR, mmHg)	76.00 [68.00, 84.00]	75.00 [70.00, 85.00]	79.50 [70.00, 83.50]	0.785
Smoke, n (%)	43 (43.0)	52 (32.7)	29 (33.7)	0.985
ALT (IQR, u/mL)	24.00 [20.00, 30.00]	31.00 [23.00, 35.00]	38.50 [32.00, 44.00]	<0.001
AST (IQR, u/mL)	32.00 [27.75, 35.00]	25.00 [20.50, 32.00]	36.50 [29.25, 43.00]	<0.001
CRP (IQR, mg/dL)	7.00 [5.00, 8.00]	9.00 [6.00, 12.00]	11.00 [8.00, 14.00]	0.001
TC (IQR, mmoL/L)	4.39 [3.68, 5.15]	4.40 [3.78, 5.15]	5.56 [4.79, 6.43]	0.06
TG (IQR, mmoL/L)	1.52 [1.1, 2.56]	1.61 (1.23, 2.22)	1.58 (1.13, 1.86)	0.11
LDL-c (IQR, mmoL/L)	2.50 [1.94, 3.16]	2.67 [1.98, 3.31]	3.45 [2.48, 4.30]	<0.001
eGFR (IQR, mL/min/1.73 $m^{2}$)	93.00 [82.75, 103.00]	92.00 [83.50, 100.00]	82.00 [68.00, 93.75]	<0.001
RBP4 (IQR, μg/mL )	45.00 [30.00, 56.00]	45.50 [35.00, 56.83]	65.00 [54.00, 71.00]	<0.001
NT-proBNP (IQR, pg/mL)	NA	278.00 [110.00, 450.00]	455.00 [130.00, 760.00]	<0.001
HbA1c (IQR, %)	NA	7.60 [6.70, 8.50]	7.70 [6.90, 8.70]	0.053
LVEF (IQR, %)	65 [61, 67]	55 [45, 66]	48 [45, 56]	<0.001
peak E velocity (cm/s)	80 [60, 95]	80 [70, 100]	70 [64, 90]	<0.001
peak A velocity (cm/s)	70 [50, 80]	65 [52, 79]	45 [40, 87]	<0.001
E/A velocity ratio	1.2 [0.7, 1.4]	1.4 [0.9, 1.6]	1.7 [1.55, 2.1]	<0.001
e’ (medial mitral annulus, cm/s)	15 [12, 18]	12 [7, 14]	10 [7, 12]	<0.001
E/e’	7 [5, 10]	10 [6, 11]	13 [12, 18]	<0.001
Left atrial volume index (mL/$m^{2}$)	23 [20, 35]	27 [22, 38]	35 [28, 45]	<0.001
Diabetes duration (IQR, years)	NA	7.00 [5.00, 11.50]	12.00 [9.25, 15.00]	<0.001
Diabetic retinopathy n (%)	NA	36 (22.6)	31 (36)	0.03
Diabetic nephropathy n (%)	NA	27 (17)	18 (20.9)	0.49
Diabetic neuropathy n (%)	NA	35 (22)	32(37.2)	0.02
Oral medication, n (%)	NA	145 (91.2)	81 (94.2)	0.558
Insulin therapy, n (%)	NA	88 (55.3)	24 (27.9)	<0.001

Data were presented as median (interquartile range) or n (%). BMI, body mass index; SBP, systolic blood pressure; DBP, diastolic blood pressure; HbA1c, hemoglobin A1c; ALT, alanine aminotransaminase; AST, aspartate aminotransaminase; CRP, C-reactive protein; TC, total cholesterol; TG, triglyceride; LDL-C, low-density lipoprotein cholesterol; eGFR, estimated glomerular filtration rate; LVEF, left ventricular ejection fraction; RBP4, retinol binding protein 4; NT-proBNP, N terminal-pro hormone BNP.

### 3.2 Risk of DCM According to Tertile of Serum RBP4 and NT-ProBNP Levels in Patients with Diabetes

The prevalence of DCM among the tertile of RBP4 were 9.8%, 32.0%, and 61.4%, respectively. The OR of DCM were increased in patients with ascending tertile of RBP4 ($P_{\text{trend}}$ < 0.001). After adjusting for gender, age, BMI, SBP, DBP, smoke, HbA1c, log NT-proBNP (for RBP4 only), OR (95% CI) associated with the tertile of RBP4 was 16.87 (6.58–43.23) ($P_{\text{trend}}$ < 0.001) (Table 2). The prevalence of DCM among the tertile of NT-proBNP were 29.3%, 23.5%, and 52.4%, respectively. The OR of DCM were increased in patients with ascending tertile of NT-proBNP ($P_{\text{trend}}$ = 0.005). After adjusting for gender, age, BMI, SBP, DBP, smoke, HbA1c, OR (95% CI) associated with the tertile of NT-proBNP was 2.13 (1.09–4.16) ($P_{\text{trend}}$ = 0.018) (Table 2).

**Table 2.** **Risk of diabetic cardiomyopathy according to tertiles of serum retinol binding protein 4 and NT-proBNP levels in patients with diabetes**.

	RBP4 (ug/mL)				NT-proBNP (pg/mL)				$P_{\text{ROC}}$
	<45	45–59.8	>59.8	$P_{\text{trend}}$	<170	170–440	>440	$P_{\text{trend}}$	
No. of DCM cases (%)	82 (33.47)	75 (30.61)	88 (35.92)		81 (33.06)	74 (30.2)	90 (36.73)		
Unadjusted OR	1.00	4.97 (1.99, 12.41)	16.7 (6.92, 40.72)	<0.001	1.00	0.80 (0.39, 1.63)	2.37 (1.25, 4.48)	0.005	
Adjusted OR									
Model $1^{a}$	1.00	4.49 (1.76, 11.41)	15.78 (6.38, 39.01)	<0.001	1.00	0.78 (0.38, 1.62)	2.26 (1.18, 4.32)	0.009	
Model $2^{b}$	1.00	4.27 (1.64, 11.08)	16.87 (6.58, 43.23)	<0.001	1.00	0.64 (0.30, 1.37)	2.13 (1.09, 4.16)	0.018	
AUROC	0.63 (0.57, 0.69)				0.80 (0.75, 0.85)				<0.001

^a^Model 1, adjusted for Gender, Age, Log NT-proBNP (for RBP4 only). ^b^Model 2, adjusted for Gender, Age, BMI, SBP, DBP, Smoke, HbA1c, Log NT-proBNP (for RBP4 only). AUROC, area under the receiver operator characteristic curve; PROC, *p* value for the comparison of area under; ROC, curves for RBP4 and NT-proBNP to predict DCM.

**Fig. 3.**
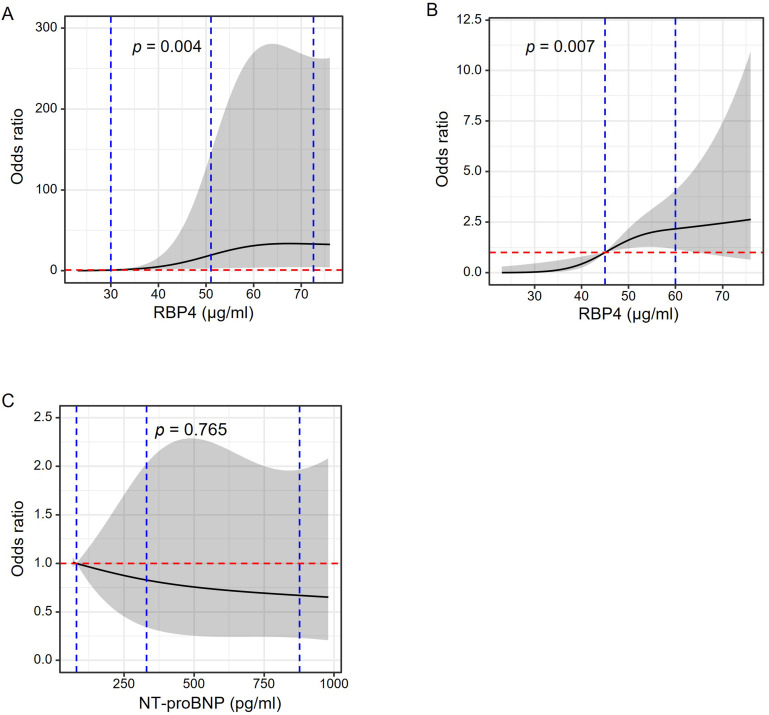
**RCS to evaluate RBP4 and NT-proBNP levels with the risk of DCM in diabetes**. Odds ratio (OR) and 95% confidence interval (CI) were derived from restricted cubic spline regression adjusted for age, gender, diabetes duration, body mass index, insulin treatment, left ventricular ejection fraction, triglyceride, low-density lipoprotein cholesterol (LDL-C), estimated glomerular filtration rate (eGFR), diabetic retinopathy, diabetic nephropathy, diabetic neuropathy and Log NT-proBNP (for RBP4 only), with knots placed at the 10th, 50th, and 90th percentiles (A) or tertiles (B) of RBP4 and tertiles of NT-proBNP (C). Blue vertical dashed lines in panel A indicate RBP4 knot cut-offs placed at 10th (30 μg/mL), 50th (51 μg/mL), and 90th (72.55 μg/mL). Blue vertical dashed lines in panel B indicate RBP4 knot cut-offs placed at tertiles (45 μg/mL, 59.8 μg/mL). Blue vertical dashed lines in panel C indicate NT-proBNP knot cut-offs placed at 10th (80 μg/mL), 50th (330 μg/mL), and 90th (877 μg/mL). Red dashed horizontal line indicates OR at 1.00. The black line indicated OR, and the shadow indicated 95% CI. *p* values were based on the likelihood ratio test.

There is significant difference of AUROC between RBP4 and NT-proBNP for diagnose DCM of diabetes (*p <* 0.001) (Table 2). We used restricted cubic splines to evaluate the pattern of association between RBP4 and NT-proBNP levels with the risk of DCM. As shown in Fig. 3, we observed a positive association of RBP4 with the risk of DCM (Fig. 3A: the likelihood ratio test reveals *p* for linearity equal to 0.004 with knots at 10th, 50th, and 90th of RBP4 levels, Fig. 3B: *p* for linearity equal to 0.007 with knots at tertiles of RBP4 levels). In contrast, we did not observe significant association between NT-proBNP and DCM risk (Fig. 3C: the likelihood ratio test reveals *p* for linearity equal to 0.765).

### 3.3 Improved Discriminative Ability and Reclassification by RBP4

We evaluated whether RBP4 improved the discriminative ability for DCM beyond other risk factors, including clinically relevant factors and significant covariates based on the univariate analyses. Adding log RBP4 to a basic risk model including age, BMI, diabetes duration, LVEF, insulin treatment, TG, LDL-C, eGFR, CRP, log NT-proBNP, retinopathy, nephropathy and neuropathy improved the c-index from 0.91 to 0.94 (*p* = 0.024) (Table 3). Improvement in reclassification by adding log RBP4 to the basic model was evaluated by NRI and IDI. With risk thresholds at 0.3 and 0.7, low, medium, and high-risk categories were defined as having <30%, 30%–70%, >70% probability of having DCM in DM patients. For continuous risk probability, a continuous NRI (95% CI) of 87.86% (64.4%–111.32%) (*p *< 0.05) indicated that the new model (basic + RBP4) improved the percentage of correct reclassification compared to the old model (basic model) by 87.86%. For ordered categorical risk probability, a categorical NRI (95% CI) of 15.07% (4.48%–25.66%) (*p *< 0.05) indicated that the new model (basic + RBP4) improved the percentage of correct reclassification compared to the old model (basic model) by 15.07%. In other words, the accuracy of the prediction of the new model with one additional predictor variable (RBP4) was increased and the new model was better than the old model.

**Table 3.** ** Reclassification and discrimination statistics for diabetic cardiomyopathy by serum RBP4 in patients with diabetes mellitus. Patients were divided into 3 risk categories: <30%, 30%–70%, >70%**.

Model	C-index		Continuous ${\mathrm{NRI}}^{b}$, %		Categorical ${\mathrm{NRI}}^{c}$		${\mathrm{IDI}}^{d}$	
	Estimate (95% CI)	*p* value	Estimate (95% CI)	*p* value	Estimate (95% CI), %	*p* value	Estimate (95% CI), %	*p* value
${\mathrm{Basic}}^{a}$	0.91 (0.88–0.95)		Ref		Ref		Ref	
${\mathrm{Basic}}^{a}$ + log RBP4	0.94 (0.91–0.97)	0.024	87.86 (64.4–111.32)	<0.001	15.07 (4.48–25.66)	0.005	7 (3–10)	<0.001

^a^Basic: Gender, Age, BMI, SBP, DBP, Smoke, TC, TG, LDL-C, HbA1c, LVEF, eGFR, log NT-proBNP, CRP, Insulin therapy, diabetic retinopathy, diabetic nephropathy, diabetic neuropathy. ^b^NRI, net reclassification improvement. ^c^Risk threshold: 0.3, 0.7. ^d^IDI, integrated discrimination improvement.

IDI stands for the difference between mean value of the predicted probability of DCM for each individual in the new model and the old model. An IDI (95% CI) of 7% (3%–10%) showed that the new model (basic + RBP4) improves predictive power by 7% over old model (basic model) (*p *< 0.05) (Table 3).

## 4. Discussion

In this study, we found that levels of RBP4 were elevated in patients with DCM. Higher serum RBP4 was independently associated with the risk of DCM. The addition of RBP4 improved the reclassification and discrimination of a DCM risk model.

DCM often accompanies other comorbidities such as obesity, dyslipidemia, and vascular disease. In the early stages, only sub-structural changes in cardiomyocytes are present. Furthermore, identifying DCM before cardiac dysfunction exacerbates may provide a critical window of time for early intervention. Computed tomography (CT), magnetic resonance imaging (MRI), and echocardiography are commonly used to detect DCM. CT is helpful because it collects end-systolic and end-diastolic volumetric data that can be reconstructed by automated software, which collects small segments of data along several cardiac cycles to produce the final image of the computed tomography. Consequently, this approach yields parameters of the ventricular function that are instrumental in the diagnosis of DCM. However, radiation exposure and the side effects associated with the use of contrast media may limit this methodology. MRI operates with a greater spatial and temporal resolution to evaluate chamber size, left ventricular EF, and myocardial mass distribution. MRI also provides extra information about information like myocardial fibrosis and subclinical ischemia [7]. However, MRI also has some limitations. MRI may underestimate diastolic dysfunction, is not compatible with some pacemakers or implantable defibrillators, and may produce claustrophobia in some patients. Thus, compared to the two methods noted above, ultrasound has obvious advantages. It uses no radiation, no contrast medium, and is widely used in clinical evaluations of cardiac function. However, but the early period of cardiac dysfunction is very difficult to detect without the TDI model at exercise stress [23]. Therefore, in our study, we chose patients with LVEF <50% or moderate to severe diastolic dysfunction.

Pathological diagnosis of the myocardium is a reliable assessment of DCM. Pathophysiological features of DCM include accumulation of advanced glycation end products (AGE), cardiomyocyte apoptosis, autophagy, myocardial fibrosis, endothelial dysfunction, left ventricular hypertrophy, and endoplasmic reticulum stress [24, 25, 26]. However, the clinical practice requires sensitive but reliable markers that can be obtained non-invasively and that accurately predict underlying disease and its severity. Several efforts have been made to improve DCM detection by quantification of biomarkers [27, 28, 29]. Our findings show that RBP4, a new adipocytokine, is a useful diagnostic marker of DCM, and circulating RBP4 was valuable in predicting the presence of DCM in diabetics. These findings provide indirect evidence of RBP4 involvement in cardiac remodeling and bring new insights into the pathophysiological role of RBP4 which might be a promising therapeutic target for DCM.

Several possible explanations could explain the association between RBP4 and DCM. Firstly, RBP4 is a novel polypeptide ligand that has been shown to play a pivotal role in the regulation of glucose homeostasis and lipid metabolism [30]. A Clinical study showed that serum RBP4 levels <31 μg/mL and RBP4 levels >55 μg/mL were associated with DM [13]. Also, transgenic overexpression of human RBP4 or injection of recombinant RBP4 in normal mice causes insulin resistance [31]. So RBP4 may involve in the development of diabetes. Secondly, elevated RBP4 in cardiac hypertrophy may have pathophysiological consequences because RBP4 increased cell size, enhanced protein synthesis, and elevated the expression of hypertrophic markers including NP precursor A (NPPA), NPPB genes, and Myh7 in primary cardiomyocytes by activating the TLR4/MyD88 pathway [32], and the onset of heart failure is typically preceded by cardiomyocyte hypertrophy. So RBP4 can induce heart failure associated with cardiomyocyte hypertrophy. Thirdly, RBP4 is related to heart development [33]. Reducing embryonic RBP4 levels can alleviate cardiac defects in zebrafish embryos [34]. Therefore, RBP4 may affect cardiac function by regulating the differentiation of cardiomyocytes. Fourthly, RBP4 promotes inflammatory damage to cardiac myocytes [13], and increased RBP4 concentration was shown to be proportional to interleukin-8 (IL-8) levels in patients with inflammatory dilated cardiomyopathy [35]. Thus, RBP4 may affect inflammatory pathways to regulate cardiac function. CRP, as a marker of inflammatory, there are significant difference of CRP levels between DCM and NDCM Which indicate CRP is partly responsible for the increasing of RBP4 in NDCM, but after adjustment of conventional risk factors, RBP4 is also independent predictor for DCM. Lastly, as we all know, many reports [36, 37] show that complication of diabetes, like retinopathy, nephropathy have a close relationship with serum levels of RBP4, in our study, after logistic regression, controlled by diabetes complication, RPB4 still is the risk factor of NDCM, which show RBP4 is clinical valuable marker for diagnoses for DCM in diabetes.

One important finding of our study is that the duration of diabetes is an independent factor for the risk of DCM. Diabetes duration was a recognized risk factor for diabetic complications in diabetic subjects [38] and the presence of AGE deposition in the hearts of patients was related to the duration of diabetes [39]. We did not find a significant correlation between RBP4 and FBG in diabetes patients and Fedders R *et al*. [40] reported that increasing circulating RBP4 did not affect glucose homeostasis in mice with liver-specific overexpression of RBP4. This result suggests that RBP4 is not always associated with glucose levels. In our study, insulin therapy may be the reason for the result between glucose and RBP4. Also, we find that insulin use in the DCM group is lower than in the NDCM group, which indicate that partially reason of DCM in diabetes was related with insulin use. Animal studies in the low-dose streptozotocin-induced diabetic rat show that markers of diabetic cardiomyopathy were markedly ameliorated following insulin replacement indicating that insulin replacement can reduce complications of diabetes including cardiomyopathy [41]. Therefore, our research provides a little support for clinical prevention of diabetic cardiomyopathy. It is necessary to investigate effect of the insulin use on DCM patients in the future.

According several other studies [42, 43, 44], they show a positive correlation between RBP4 and LDL-C. Our result shows that BMI was not significantly different among the groups, because correlation analysis showed that RBP4 was positively correlated with BMI [42]. To minimize the effect of BMI on RBP4 concentrations, we calculated the reclassification and discrimination for DCM by serum of RBP4 in patients with DM, which show RBP4 is a valuable marker for the risk of DCM. RBP4 is cleared from the circulation by the kidneys [45]. Decreasing eGFR was associated with higher levels of RBP4 in hypertension [42]. RBP4 increased in DCM was associated with reducing renal clearance, rather than increasing secretion of adipocytes, which might also account for our finding. When controlled with eGFR, diabetic nephropathy and other parameters, we still found RBP4 is the risk factor for DCM in patients with diabetes. Thus, renal dysfunction is not enough to explain the higher RBP4 concentrations in DCM. Although age and gender were shown in other studies to influence the levels of RBP4 [46, 47, 48], in our study the age of DCM group has a higher level than NDCM group, in order to eliminate this interference factor, we adjusted HR by multivariate logistic regression analyses, the RBP4 level was independently predictive of DCM in diabetes.

Our study has several limitations. First, this is a case-control study that could not establish the causative role of RBP4 in DCM prediction. Secondly, the sample size was relatively small. Our study enrolled diabetic patients with moderate and severe diastolic dysfunction, the mild diastolic dysfunction of diabetes which accounts for more diabetic samples did not include. This design can better ensure more reliable conclusions. Thirdly, we could not follow up with incident DCM with only a single echocardiographic evaluation. We will address these points with a larger prospective cohort in our future studies.

## 5. Conclusions

This study investigated the RBP4 levels in DM patients. We found that serum RBP4 levels were higher in DCM patients than in DM patients without DCM. Moreover, the elevated serum levels of RBP4 are associated with the risk of DCM in patients with DM. The results suggested the role of RBP4 was a potential biomarker for the diagnosis of DCM in DM.
